# Fear of childbirth and its predictors in re-pregnant women after cesarean section: a cross-sectional multicenter study in China

**DOI:** 10.1186/s12884-022-04721-z

**Published:** 2022-05-07

**Authors:** Yiping Hou, Xihong Zhou, Min Yao, Sai Liu

**Affiliations:** 1 Clinical Nursing Teaching and Research Section, The Second Xiangya Hospital and Xiangya School of Nursing, Central South University, Changsha, Hunan China; 2 Clinical Nursing Teaching and Research Section and Department of Obstetrics, The Second Xiangya Hospital, Central South University, Changsha, Hunan China

**Keywords:** Fear of childbirth, Predictors, Re-pregnant women, Cesarean section, Childbirth self-efficacy, Social support

## Abstract

**Background:**

Since the implementation of China’s two-child policy in 2016, the number of re-pregnant women after cesarean section has increased significantly. These women are more prone to fear of childbirth compared with primiparas due to their history of scarred uterus leading to a more complicated delivery process, which poses a great threat to their physical and mental health. However, there is currently limited research on the problem in China. The aim of this study was to assess fear of childbirth and its predictors in re-pregnant women after cesarean section in China.

**Methods:**

A cross-sectional multicenter study was conducted in three hospitals from June 7 to December 7, 2020, in Changsha, China. Study hospitals were selected using a random sampling technique. Participants were selected using a convenience sampling technique. Three hundred fifty-eight women during the third trimester of pregnancy who were older than 18 years, having a history of CS(s), and not having major physical or mental health problems were included. Fear of childbirth and its predictors were evaluated using the Childbirth Attitude Questionnaire, the short form of the 32-item Chinese Childbirth Self-Efficacy Inventory, the Social Support Rating Scale, and the demographic-obstetric data sheet. After checking for completeness, data were exported to statistical software for analysis. Both univariate analysis and multiple linear regression analysis were computed to assess fear of childbirth and its predictors. Statistical significance was declared at a *P*-value of < 0.05.

**Results:**

The average score of fear of childbirth was 43.76 (standard deviation = 5.27, range 17–58). Number of cesarean sections, experience with previous cesarean section, childbirth self-efficacy and social support were significantly associated with fear of childbirth (*P* < 0.05).

**Conclusions:**

In this study, re-pregnant women after cesarean section in China had moderate fear of childbirth, and the number of cesarean sections, experience with previous cesarean section, childbirth self-efficacy and social support were predictors of fear of childbirth. It is important for healthcare professionals to find re-pregnant women after cesarean section at high risk of fear of childbirth and provide appropriate services during pregnancy.

## Background

Fear of childbirth (FOC) is described as a negative feeling before, during or after delivery when thinking about future delivery or experiencing others’ fearful responses to childbirth and labor pain [1]. In other words, FOC is a negative cognitive assessment of the pathological dread and avoidance of childbirth [2] and manifests in the form of physical discomfort, nightmares, and difficulty concentrating on work and family activities [3]. In addition, FOC may develop during pregnancy, and the content of such fear may include pain, uncertainty, loss of control, and the possibility of having an impaired or stillborn child [4]. It is not uncommon for pregnant women to fear childbirth, as it has been estimated that FOC affects approximately 14% of pregnant women worldwide [5]. Studies in China have also shown that the incidence of FOC among women is 50% or higher [6]. The degree of FOC varies from person to person, ranging from mild to severe fear. Research has reported that 20% of pregnant women experience moderate FOC and that 6-13% of pregnant women experience severe FOC [7].

FOC has many negative effects on women’s physical and mental health, including pregnancy complications, severe pain and the use of anesthesia during childbirth, prolonged childbirth, mother-child relationship difficulties, postpartum depression and posttraumatic stress disorder [7–13]. In addition, FOC is often one of the factors for pregnant women to request a cesarean section (CS), which may lead to the occurrence of a CS without medical indications [4], resulting in a waste of medical resources and increasing socioeconomic burdens (In China, under the premise that the obstetrician fully explains the delivery mode, pregnant women still have the right to choose the delivery mode). Increasing evidence has shown that FOC is related to the mode of delivery [14, 15]. A large cohort study of more than 700,000 pregnant women found that the CS rate of women with FOC was 4.4 times higher than that of women without FOC [12]. Studies have indicated that young, low-educated women are more likely to suffer from FOC compared to older, high-educated women [16]. In China, high obstetric intervention rates and the lack of high-quality maternal care may cause pregnant women to fear childbirth. Fear of pain and a previous difficult delivery have also been found to be related to FOC [17].

Since the implementation of China’s two-child policy in 2016, the number of pregnant women has increased significantly [18]. The high rate of CS (40-60%) in the past has increased the number of re-pregnant women after CS [19]. These women are a special group compared to primiparas due to their history of scarred uterus leading to a more complicated delivery process, which makes them more incline to FOC [20]. Moreover, these women not only have the same FOC as that of primiparas but also the fear that their previous CS may cause various complications in their current pregnancy and childbirth: postpartum hemorrhage, infection, placenta previa, and placenta accreta [21].

Against the special background of the two-child policy and high CS rates in China, it is important for healthcare providers to understand, recognize and address FOC in re-pregnant women after CS. Although this problem requires urgent attention, to the best of our knowledge, in China, (1) most studies have focused on anxiety and depression, while the research on FOC is limited; (2) studies have concentrated on primiparas and have often ignored re-pregnant women after CS; and (3) furthermore, the available studies on FOC have been conducted in high-income areas, and thus, little is known about this problem in low-income areas. Given the limited understanding of FOC in re-pregnant women after CS in China, this study aimed to assess FOC and its predictors.

## Methods

### Study design and setting

A cross-sectional descriptive study was conducted from June 7 to December 7, 2020, in Changsha, China. Changsha is the provincial city of Hunan province located in the central-south of China, with a population of approximately 8 million. The birth rate in Changsha is 12.43‰ (12.43 births for every 1000 people per year). A simple random sampling technique by picking the number assigned to each hospital from a box was used to select the three general and specialty hospitals out of 10 utilized for data collection. These hospitals (The Second Xiangya Hospital, Hunan Provincial People’s Hospital and Changsha Maternal and Child Health Hospital) were chosen because they provide antenatal services and obstetrics care. The annual delivery rate of each hospital is approximately 4000 live births. The sample was apportioned to every hospital by investigating the number of deliveries earlier in this year until the complete sample was obtained.

### Sample size determination and participants

The required sample size was determined using a single population formula based on the assumption of a 95% confidence interval with a margin of error of 5%. Women during the third trimester of pregnancy who were receiving antenatal care at the study hospitals during the study period were recruited using a convenience sampling technique. The inclusion criteria were as follows: (1) being older than 18 years, (2) having a history of CS(s); (3) being able to understand the content of the questionnaires, and (4) providing consent to participate. Women with concurrent major physical or mental health problems were excluded.

### Materials

In this study, the Childbirth Attitude Questionnaire (CAQ), which was developed by Lowe in 2000 [22], was used to measure FOC. The CAQ is a 16-item questionnaire that uses a 4-point Likert scale. The item scores are summed to provide a total score (range 16-64), with higher scores indicating higher levels of FOC. The Chinese version of the CAQ has been used with good reliability and validity in pregnant Chinese women in 2016 [23]. The Cronbach’s alpha for the scale is 0.91. For the classification of no, low, moderate and severe levels of FOC, the cutoff values are 16, 28, 40, and 52, respectively.

The short form of the 32-item Chinese Childbirth Self-Efficacy Inventory (CBSEI-C32) [24] was used in this study to measure childbirth self-efficacy. The scale has two parallel subscales—the outcome expectancy subscale and the efficacy expectancy subscale—which consist of the same 16 items measuring coping behavior for childbirth on a 4-point Likert scale. The sum of each subscale is the total score (range 32-128), with higher scores indicating higher levels of childbirth self-efficacy. The scale has a reported Cronbach’s alpha value of 0.96 and has been shown to be a valid and reliable measure in Mainland China in 2011 [24].

The Social Support Rating Scale (SSRS), which was developed by Xiao [25], was used to measure social support in this study. There are 10 items in this scale, including three dimensions—objective support (3 items), subjective support (4 items) and the utilization of social support (3 items). The sum of each dimension is the total score, with higher scores indicating higher levels of social support. The scale has Cronbach’s alpha values of 0.896 and has been used widely in China in 1987 [25].

Demographic and obstetric data included age (< 35/≥35), pregnant women who were the only child in their families (no/yes), residential area (rural/urban), education (elementary school and below/junior high school/senior high school/college and above), occupation (office clerk/agricultural worker/self-employed/freelance), self-rated economic status (poor/fair/good), number of CSs (1/≥2), and experience with previous CS (no or mild fear/moderate or severe fear).

### Procedures and ethical consideration

Ethical approval was obtained from the ethics committee of each hospital. Participation in the study was voluntary, and all participants provided written informed consent before participation.

After obtaining ethical approval, a pilot study was conducted with 10 eligible subjects to evaluate the feasibility of the study and identify any unpredictable data collection problems, and no problems were reported. Training for data collectors was provided to ensure the accuracy and consistency of data collection. All eligible subjects who were waiting for their appointment in the obstetric clinic of the research hospitals were invited to participate in the study. After providing informed consent, participants were asked to complete the demographic and obstetric questionnaire, the CAQ, the CBSEI-C32 and the SSRS. Data collectors remained in the vicinity to answer questions and personally collected the returned questionnaires.

### Data processing and analysis

After data collection, all collected questionnaires were checked for completeness and internal consistency to exclude missing or inconsistent data. Data were entered and analyzed using IBM SPSS for Windows, version 16.0 (SPSS Inc., Chicago, IL, USA). Mean (M), standard deviation (SD), frequency and percentage were used to describe demographic and obstetric data, CAQ, CBSEI-C32 and SSRS scores. Independent sample *t-*test and one-way analysis of variance were used to compare the CAQ scores with different demographic and obstetric characteristics. Pearson correlation coefficients were calculated to measure the relationships among CBSEI-C32, SSRS, and CAQ. If *P* < 0.05 in the above tests, then significant variables were entered in the multiple linear regression analysis to predict FOC. The variance inflation factor (VIF) was used to assess multicollinearity among the predictors.

## Results

### Sample and demographic characteristics of participants

A total of 410 eligible women visited the clinics during the period of investigation. Forty-one women (10%) refused to participate and 11 women (2.68%) consented but did not complete the questionnaires, resulting in a total of 358 pregnant women being included in this study. The ages of these women ranged from 22 to 44 years (M = 33.60, SD = 4.02), and their M gestational age was 253.82 days (SD = 17.93) at enrollment. All the participants were married.

### CAQ scores

The average CAQ score among these re-pregnant women was 43.76 (SD = 5.27, range 17-58). The items with high scores were “I have a fear of my baby being injured during childbirth” (M = 3.42, SD = 0.79) and “I have a fear of painful labor contractions” (M = 3.41, SD = 0.78) (Table 1).

**Table 1:** CAQ scores listed by item (*n* = 358)

Original item no.	CAQ items	M (SD)
11	I have a fear of my baby being injured during childbirth.	3.42 (0.79)
12	I have a fear of painful labor contractions.	3.41 (0.78)
9	I have a fear of needing a cesarean section.	3.40 (0.79)
6	I have some fear of something being wrong with my baby.	3.31 (0.88)
2	I am truly afraid of giving birth.	3.19 (0.76)
16	Overall, I would rate my anxiety about childbirth as 1 (no anxiety), 2 (low anxiety), 3 (moderate anxiety), or 4 (high anxiety).	3.16 (0.77)
10	I have a fear of being torn during the birth of my baby.	2.95 (0.82)
4	I have a fear of bleeding too much during childbirth.	2.89 (0.84)
1	I have a fear of losing control of myself during childbirth.	2.71 (0.89)
13	I have difficulty relaxing when thinking of the upcoming birth.	2.54 (0.66)
7	I have a fear of painful injections.	2.33 (0.57)
8	I have a fear of being left alone during labor.	2.22 (0.71)
5	I have a fear that I will not be able to help during childbirth.	2.16 (0.72)
15	I have a fear of not getting the kind of care that I want.	2.13 (0.81)
14	I have a fear of the hospital environment.	2.08 (0.58)
3	I have nightmares about childbirth.	1.85 (0.65)

*Abbreviations*: *CAQ* Childbirth Attitude Questionnaire, *M* mean, *SD* standard deviation

### Demographic and obstetric characteristics and their association with the CAQ

Of the pregnant women studied, 72.91% (*n* = 261) were younger than 35 years, and 32.96% (*n* = 118) were only child in their families. The majority (63.69%, *n* = 228) of the women lived in urban areas, and 14.81% (*n* = 53) had an educational level of college or above. Approximately 36.03% of respondents (*n* = 129) were office clerks, and 49.16% (*n* = 176) reported good economic status. The minority (8.66%, *n* = 31) of these women had more than 2 CS events, and 33.8% (*n* = 77) had moderate or severe fear of their previous CS. Pregnant women who were the only child in their families, self-rated economic status, number of CSs, and experience with previous CS were significantly associated with the CAQ (*P* < 0.05) (Table 2).

**Table 2:** Demographic and obstetric characteristic and comparisons of CAQ scores among subgroups (*n* = 358)

Variables	Total n (%)	CAQ M (SD)	***F*** or ***t***	***P***
Age (years)			−1.81	0.071
< 35	261 (72.91)	43.46 (5.30)		
≥ 35	97 (27.09)	44.59 (5.13)		
The only child in her family			3.42	0.001
No	240 (67.04)	43.10 (5.36)		
Yes	118 (32.96)	45.10 (4.85)		
Residential area			0.13	0.894
Rural	130 (36.31)	43.81 (4.18)		
Urban	228 (63.69)	43.74 (5.82)		
Education			1.82	0.143
Elementary school and below	39 (10.89)	42.69 (4.87)		
Junior high school	60 (16.76)	43.63 (7.04)		
Senior high school	206 (57.54)	43.65 (4.80)		
College and above	53 (14.81)	45.15 (4.85)		
Occupation			1.69	0.168
Office clerk	129 (36.03)	44.12 (4.58)		
Agricultural worker	13 (3.63)	43.00 (4.60)		
Self-employed	89 (24.86)	42.74 (6.46)		
Freelance	127 (35.48)	44.19 (5.01)		
Self-rated economic status			4.16	0.006
Poor	41 (11.45)	46.34 (5.08)		
Fair	141 (39.39)	43.78 (5.13)		
Good	176 (49.16)	43.15 (5.27)		
Number of CSs			−3.60	0.000
1	327 (91.34)	43.46 (5.15)		
≥ 2	31 (8.66)	46.97 (5.54)		
Experience with previous CS			−3.37	0.001
No or mild fear	281 (66.20)	43.28 (5.36)		
Moderate or severe fear	77 (33.80)	45.53 (4.55)		

*Abbreviations*: *CAQ* Childbirth Attitude Questionnaire, *M* mean, *SD* standard deviation, *CS* cesarean section

### Correlations among the CBESI-C32, SSRS, and CAQ

The average total objective value of the CBSEI-C32 among respondents was 87.59 (SD = 18.81, range 32-128). The average total SSRS score among these women was 45.35 (SD = 7.97, range 17-60). Pearson correlation coefficients indicated that CBSEI-C32 and SSRS were significantly negatively related to the CAQ (*P* < 0.01) (Table 3).

**Table 3:** Relationships among the CBSEI-C32, the SSRS, and CAQ scores (*n* = 358)

Variable	CAQ	CBSEI-C32	SSRS
CAQ	1	−0.905**	−0.869**
CBSEI-C32	−0.905**	1	0.979**
SSRS	−0.869**	0.979**	1

*Abbreviations*: *CAQ* Childbirth Attitude Questionnaire, *CBSEI-C32* 32-item Chinese Childbirth Self-Efficacy Inventory, *SSRS* Social Support Rating Scale

^**^p < 0.01

### Multiple linear regression analysis of FOC

Based on the significant results between candidate predictors and the FOC, these variables (pregnant women who were the only child in their families, self-rated economic status, number of CSs, experience with previous CS, childbirth self-efficacy, and social support) were validated through multiple linear regression (Table 4), which showed that number of CSs, experience with previous CS, childbirth self-efficacy and social support were significantly associated with the CAQ (*P* < 0.05). Among the predictors, childbirth self-efficacy had the most effect (Beta = − 1.284). The VIF value was less than 10, which indicated that there was no multicollinearity among the variables. These variables accounted for 83.1% of the total variance. The analysis was found to be statistically significant (*F* = 440.77, *P* < 0.000) (Table 5).

**Table 4:** Independent variable assignment of factors associated with FOC

Variable	Assignment
The only child in her family	1 = no; 2 = yes
Self-rated economic status	1 = poor; 2 = fair; 3 = good
Number of CSs	1 = 1; 2 = ≥2
Experience of previous CS	1 = no or mild fear; 2 = moderate or severe fear
Childbirth self-efficacy	Original value
Social support	Original value

*Abbreviations*: *FOC* fear of childbirth, *CS* cesarean section

**Table 5:** Multiple linear regression analysis of predictors of FOC

Variable	***B***	***Beta***	***SE***	***t***	***P***	***95% CI***		VIF
						Lower	Upper	
Constant	66.905	–	1.137	58.866	0.000	64.670	69.141	–
Childbirth self-efficacy	−0.360	−1.284	0.030	−12.092	0.000	−0.418	−0.301	3.864
Number of CSs	1.333	0.071	0.449	2.969	0.003	2.217	0.450	1.218
Social support	−0.234	−0.353	0.071	−3.312	0.001	−0.095	−0.372	4.012
Experience with previous CS	0.623	0.049	0.302	2.061	0.040	1.217	0.029	1.176

*F* = 440.77, *p* < 0.000; R^2^ = 0.833, R_ad_ = 0.831

*Abbreviations*: *B* partial regression coefficient, *Beta* standard regression coefficient, *SE* standard error, *CI* confidence interval, *FOC* fear of childbirth, *CS* cesarean section, *VIF* variance inflation factor

## Discussion

We used the CAQ to assess the FOC in re-pregnant women after CS in China. The total CAQ score in our study was 43.76, which was close to that in a study conducted in Turkey [26] and higher than a study conducted in the Greece (31.22) [27]. This result indicated that re-pregnant women after CS in China might have the same moderate FOC as do women in Turkey, which was higher than that of pregnant women in the Greece. This finding may be related to different study populations. Re-pregnant women after CS in our study were more likely to have FOC due to their history of CS, leading to complicated childbirth compared with primiparas in the Greek study.

Furthermore, our study indicated that re-pregnant women after CS had the most fears for their baby’s health and painful labor contractions, a finding that was consistent with a study by Lowe et al. [22]. Therefore, the CAQ can be used to identify the specific areas of re-pregnant women’s FOC. Moreover, appropriate intervention can target the areas where re-pregnant women obtained high scores.

Our research found that the number of CSs (≥2) was positively correlated with FOC, which was consistent with a related study [20]. No research articles reporting this discovery have been published globally. A possible reason for this may be that most studies were focused on primiparas’ FOC, whereas our study included a sample of re-pregnant women with a history of CS(s). The degree of FOC in re-pregnant women after CS was higher than that of primiparous women, which may be related to the following reasons. First, a CS is a traumatic surgical procedure, and women thus bear potential risks. Experience with a previous CS affects women’s future reproductive ability and increase their risk of ectopic pregnancy and placental implantation [28]. Second, repeated CSs (≥2) without medical indications may cause uterine rupture in the perinatal period because of a scarred uterus, and pose a threat to the child [29]. Thus, clinicians should detect re-pregnant women with a history of repeated CSs at high risk for FOC and initiate or refer them to appropriate services during pregnancy.

This study showed that the experience of women with previous CS (moderate or severe fear) was positively related to FOC, which was in alignment with the results of studies conducted in Turkey and Hungary [30, 31]. The reason may be that women who had negative childbirth experience in a previous CS were worried that a similar negative experience may occur in their next childbirth experience. Previous studies showed that the occurrence of situations such as a negative birth experience and birth trauma could lead to FOC among women [32, 33]. However, Phunyammalee et al. found that a similar FOC between women with and without previous CS [34], which may be attributable to the fact that women received better care in their previous CSs, resulting in a less negative experience. Therefore, it is clear that women with negative delivery experience should receive more support from professionals during pregnancy.

Our study indicated that childbirth self-efficacy was negatively related to FOC, which was consistent with other studies [35, 36]. According to self-efficacy theory, emotional arousal is one of the sources of self-efficacy [37]. Re-pregnant women after CS tend to have negative emotions during childbirth, which may be related to their concerns about labor pains, uterine rupture, and fetal health. The arousal of disgust caused by threatening situations usually reduces self-efficacy. Moreover, previous studies have reported that self-efficacy was a determinant of FOC in pregnant women [36], indicating that improvement of the self-efficacy of pregnant women can increase their confidence during childbirth and may help reduce their FOC.

In addition to the aforementioned factors, we also observed that social support was negatively correlated with FOC a finding that was similar to those of other studies [38, 39]. Social support is an important factor in maintaining individual mental health during pregnancy. The study by Fisher et al. showed that social relationships can enhance women’s beliefs that childbirth is a physiological and controllable process, thereby improving their mental health and reducing their FOC [40]. A lack of social support or expressed dissatisfaction with one’s partner was also predictive of FOC. Furthermore, the more dissatisfied women were with their partnership and the lack of social support, the more fearful they were of childbirth [3]. Therefore, actual information about the need for more social support during pregnancy is crucial for helping pregnant women actively relieve their FOC and approach delivery.

### Strengths and limitations

This study randomly selected three hospitals in Changsha to investigate the FOC and its predictors in re-pregnant women after CS. This will help fill an important gap and raise awareness to this issue. There are several limitations of the study that should be noted. First, study hospitals accept many pregnant women from rural and mountainous areas, and there are differences in their family economic conditions, education level and culture backgrounds. Although we used regression analysis to control confounders, the above variables could potentially affect the FOC. Moreover, all participants reported marital status and harmonious spouses’ relationship in this study. Live-in partnership and discordant spouses’ relationship usually undeclared due to the relatively conservative social circumstances in China. Hence, we missed having more relevant information to assess its impact on the FOC. Second, this study was conducted in three hospitals in a particular city, so it cannot be representative of the majority of pregnant women in China. Further research is needed on women in other areas of the country.

## Conclusions and implications for practice

In this study, re-pregnant women after CS in China had moderate FOC, and the number of CSs, experience with previous CS, childbirth self-efficacy and social support were predictors of FOC. The identification of the predictors of FOC in re-pregnant women after CS in China assists in the identification of those who may have FOC. The early identification of women with FOC will allow healthcare professionals to provide appropriate interventions to reduce FOC during pregnancy, which will decrease the risk of negative psychological and obstetric consequences in these women in the future.

## Data Availability

The datasets used and/or analyzed during the current study are available from the corresponding author on reasonable request.

## Abbreviations

FOC: Fear of childbirth
CS: Cesarean section
M: Mean
SD: Standard deviation
CAQ: Childbirth Attitude Questionnaire
CBSEI-C32: 32-item Chinese Childbirth Self-Efficacy Inventory
SSRS: Social Support Rating Scale
VIF: Variance inflation factor
B: Partial regression coefficient
Beta: Standard regression coefficient
SE: Standard error
CI: Confidence interval
